# Still Searching for Better Butter Flavoring

**DOI:** 10.1289/ehp.120-a457

**Published:** 2012-12-03

**Authors:** Carol Potera

**Affiliations:** Carol Potera, based in Montana, has written for *EHP* since 1996. She also writes for *Microbe*, *Genetic Engineering News*, and the *American Journal of Nursing*.

After diacetyl (2,3-butanedione) was shown to cause bronchiolitis obliterans in workers who inhaled high levels of this volatile butter flavoring at microwave popcorn processing plants,^1^^,^^2^^,^^3^ some manufacturers switched to a substitute assumed to be safer. Recent rodent studies, however, suggest that the substitute, 2,3-pentanedione, may be as damaging to the respiratory tract as the diacetyl it replaced.^4^^,^^5^

Bronchiolitis obliterans, or “popcorn lung,” is a rare, irreversible, debilitating lung disease in which inflammation and scarring obstruct the smallest airways, the bronchioles. There is no good treatment for the disease short of a lung transplant.^6^ Ironically, lung transplant itself can trigger bronchiolitis obliterans through organ rejection mechanisms, limiting the survival of many transplant patients.^7^

After the disease was identified in popcorn workers, researchers at the National Institute for Occupational Safety and Health (NIOSH) reported bronchial epithelial damage in rats inhaling vapors of butter flavoring.^8^ This type of injury causes fibrosis (thickening and scarring) of the bronchioles, which is the underlying cause of bronchiolitis obliterans. In 2008 some of the same NIOSH researchers narrowed their studies to diacetyl, a major component of butter flavoring vapors, and reported similar acute damage in rats.^9^ The same year, Daniel Morgan, head of the Respiratory Toxicology Group at the National Institute of Environmental Health Sciences, and colleagues reported that mice develop bronchial epithelial damage after inhaling diacetyl vapors at concentrations equivalent to worker exposures.^6^ Rats proved even more susceptible than mice and developed full-blown bronchiolitis obliterans after repeated exposure to diacetyl.^4^^,^^10^

Because diacetyl and 2,3-pentanedione, both diketones, are chemically very similar, Morgan suspected that inhaled 2,3-pentanedione may be toxic to the respiratory tract as well. He exposed rats to up to 200 ppm of 2,3-pentanedione for either 10 or 12 days, using the same doses and exposure times as the earlier diacetyl rat experiments. Like diacetyl, 2,3-pentanedione caused fibrosis of the small airways.^4^^,^^7^

Morgan’s team also reported an increase in growth factors and cytokines in the lungs, including C-reactive protein and fibroblast growth factor 9.^4^ In recent unpublished studies, they observed that the gene for transforming growth factor β was highly upregulated in fibrotic airways of rats exposed to 2,3-pentanedione. This potent fibrogenic agent initiates the fibrosis pathway, causing thickening of bronchial walls and airway obstruction.^11^

Morgan’s findings provide strong evidence that inhaled diketone vapors can produce airway fibrotic disease and support a causal relationship between workplace exposure to high concentrations of diketones and the development of bronchiolitis obliterans, according to John Morris, a professor of pharmacology and toxicology at the University of Connecticut, Storrs. “Effective control strategies must be implemented to limit worker exposure to diketone vapors,” Morris says.

**Figure:**
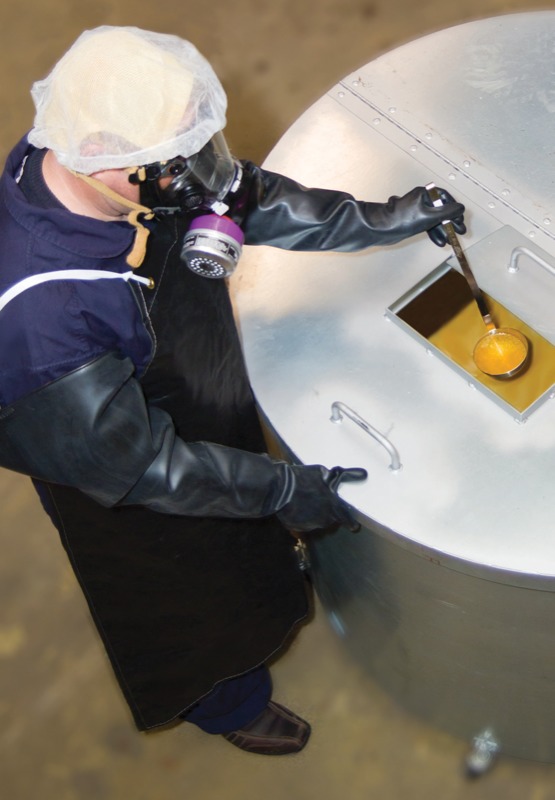
Engineering controls such as ventilated mixing vessels with hinged lids or access ports are the first line of protection against worker exposures to diketones in flavorings. Workers should also wear appropriate personal protective equipment. NIOSH

2,3-Pentanedione and diacetyl are designated as “generally recognized as safe” by the U.S. Food and Drug Administration and other qualified experts for use in foods or food packaging. However, this designation means only that they are safe to eat and does not imply safety for other routes of exposure, such as inhalation.^1^

Morgan’s data may help regulatory agencies set exposure limits to protect workers. Both NIOSH and the Occupational Safety and Health Administration currently recommend occupational flavoring control measures including isolating flavor production and handling, installing engineering controls (e.g., engineering hoods and local exhaust ventilation systems), and wearing appropriate respiratory protection.^1^^,^^12^

Additionally, the chemically induced rodent bronchiolitis obliterans model could be used to evaluate drugs that prevent progression of the disease. Transforming growth factor β offers a good drug target to block the start of the fibrosis process. “Hopefully our work will have clinical applications,” Morgan says.

Other studies indicate that 2,3-pentanedione may affect bodily systems beyond the respiratory tract. A team led by Ann Hubbs, a veterinary pathologist at NIOSH, observed not only damage to the lining of the airways but also necrosis and apoptosis in the olfactory neuroepithelium and markers of neurotoxicity in the olfactory bulb, striatum, hippocampus, and cerebellum.^5^ The functional importance of these changes in brain tissue will be addressed in future studies.

The immediate message, Hubbs says, is that “if a chemical substitution is intended to control health effects, the substitute should be demonstrably safer than the compound it replaces.”
